# The association between community-based transportation use and depressive symptoms among older adults in Japan

**DOI:** 10.1007/s44192-026-00395-7

**Published:** 2026-02-23

**Authors:** Kazushige Ide, Ryunosuke Shioya, Shuhei Kobayashi, Risa Maeda, Katsunori Kondo

**Affiliations:** 1 Center for Preventive Medical Sciences, Chiba University, 1-33 Yayoicho, Inage-Ku, Chiba-City, Chiba 263-8522 Japan; 2 Japan Agency for Gerontological Evaluation Study, Chiba, Japan; 3 Institute for Health Economics and Policy, Association for Health Economics Research and Social Insurance and Welfare, Minato-Ku, Tokyo, Japan

**Keywords:** Depressive symptoms, Frequency of outing, Social support, Public transportation, Older adults

## Abstract

**Purpose:**

The electric vehicle, called Green Slow Mobility (GSM) promoted in Japan as an age-friendly mobility solution. While interest in GSM has grown, evidence of its health impact remains limited. In Matsudo City, community residents operated the GSM service, providing an example of community-led mobility support in an urban setting. We aimed to examine associations between GSM use and health-related outcomes among older adults in Matsudo City, Japan.

**Methods:**

We analyzed two-wave longitudinal data from GSM service areas. Our analytic sample included 4,080 adults aged ≥ 65 years who completed both 2022 and 2023. We defined GSM users as those who reported using GSM at least once per month. We treated depressive symptoms (GDS-15; 0–15) as the primary outcome and frequency of outings (0–6) and the number of social supports (0–4) as secondary outcomes. We applied augmented inverse probability weighting to estimate average treatment effects (ATE), adjusting for 13 baseline covariates including sociodemographic characteristics, health indicators, social participation, and baseline values of all outcome variables.

**Results:**

GSM users reported fewer depressive symptoms (ATE = − 1.39; 95% CI: − 1.94 to − 0.83), higher frequency of outings (ATE = 0.60; 95% CI: 0.43 to 0.77), and had more social supports (ATE = 0.25; 95% CI: 0.12 to 0.38) than non-users.

**Conclusions:**

Our findings suggest that community-based transport systems such as GSM can enhance mental health and social well-being and may promote healthy aging in urban populations. Our results may inform policymakers seeking sustainable transport models that support both mobility and public health.

**Supplementary Information:**

The online version contains supplementary material available at 10.1007/s44192-026-00395-7.

## Introduction

Transportation is an important social determinant of health [1] and plays a critical role in promoting healthy aging [2, 3]. The World Health Organization identifies transportation as one of the core domains of age-friendly cities [4]. The impacts of transport systems on health are increasingly well recognized. Researchers have consistently shown that using public transportation supports a wide range of positive health-related outcomes among older adults. Studies have linked using public transportation to slower declines in walking speed [5], improved access to healthcare services [6, 7], increased physical activity [8, 9] and social engagement [10–12], better cognitive function [11], reduced risks of obesity [8, 13], depression [9, 14, 15] and loneliness [15], and lower mortality [16].

While public transportation plays a vital role in ensuring equitable mobility and supporting population health, its continuity has become increasingly uncertain [17]. A combination of demographic aging, constrained municipal budgets, and rising policy attention to climate change and carbon neutrality [18–20] has placed growing pressure on local governments. As a result, many policymakers have made difficult decisions to scale back or discontinue public transportation, particularly in areas with declining ridership or operational inefficiencies [21, 22]. This trend has raised alarm about potential negative consequences for public transportation equity, social inclusion, and healthy aging [3, 23]. As municipal governments struggle to maintain public transportation on their own, new models of mobility that involve residents, and various stakeholders have become essential.

Against this background, the electric vehicle, called Green Slow Mobility (GSM) [24] has emerged as a promising alternative to public transport. GSM refers to low speed (under 20 km/h), environmentally friendly electric vehicles that operate on public roads. GSM serves as an optimal solution for low-capacity, short-distance transport, typically supporting one-way trips of 1 to 3 km [24]. Its compact design enables smooth travel through narrow residential streets and steep terrain. Transportation planners use GSM to connect residential neighborhoods, local hubs, and transit stations—effectively bridging the first and last mile of the mobility chain. The Japanese government has promoted GSM as an adaptable and inclusive mode of transportation, particularly suited to the needs of aging communities. Notably, community-led GSM operation models offer potential for both mobility support and strengthened social capital at the local level [24–26]. In a previous short-term evaluation of GSM implementation in the community [25, 26], older users reported more favorable subjective psychosocial changes and perceived that going out had become easier compared to non-user. While policymakers are increasingly interested in GSM, evidence on its health effects remains limited.

To address this gap, we analyzed two-wave panel data from Matsudo City, an urban municipality that recently introduced GSM. Our study aimed to examine the association between GSM use and health-related outcomes. We focus on depressive symptoms, social support, and frequency of outings as key indicators, given their importance in healthy aging. Depression represents one of the leading contributors to disease burden in older adults [27], while social support and frequency of outings have been shown to play protective roles against depressive symptoms [28, 29]. By examining the role of innovative transport systems such as GSM, our findings contribute to evidence-based policymaking for healthy aging in the context of population aging.

## Methods

### Study design and setting

We conducted a follow-up study and applied a propensity score weighting (PSW) approach to estimate differences in health-related outcomes between older adults who used GSM and those who did not. We obtained data from surveys conducted in GSM service areas and from a subsample of the Japan Gerontological Evaluation Study (JAGES) [30, 31]. JAGES is a nationwide prospective cohort study investigating social determinants of health among Japanese older adults aged 65 years and older who have not been certified as requiring long-term care [30, 31].

**Fig. 1:**
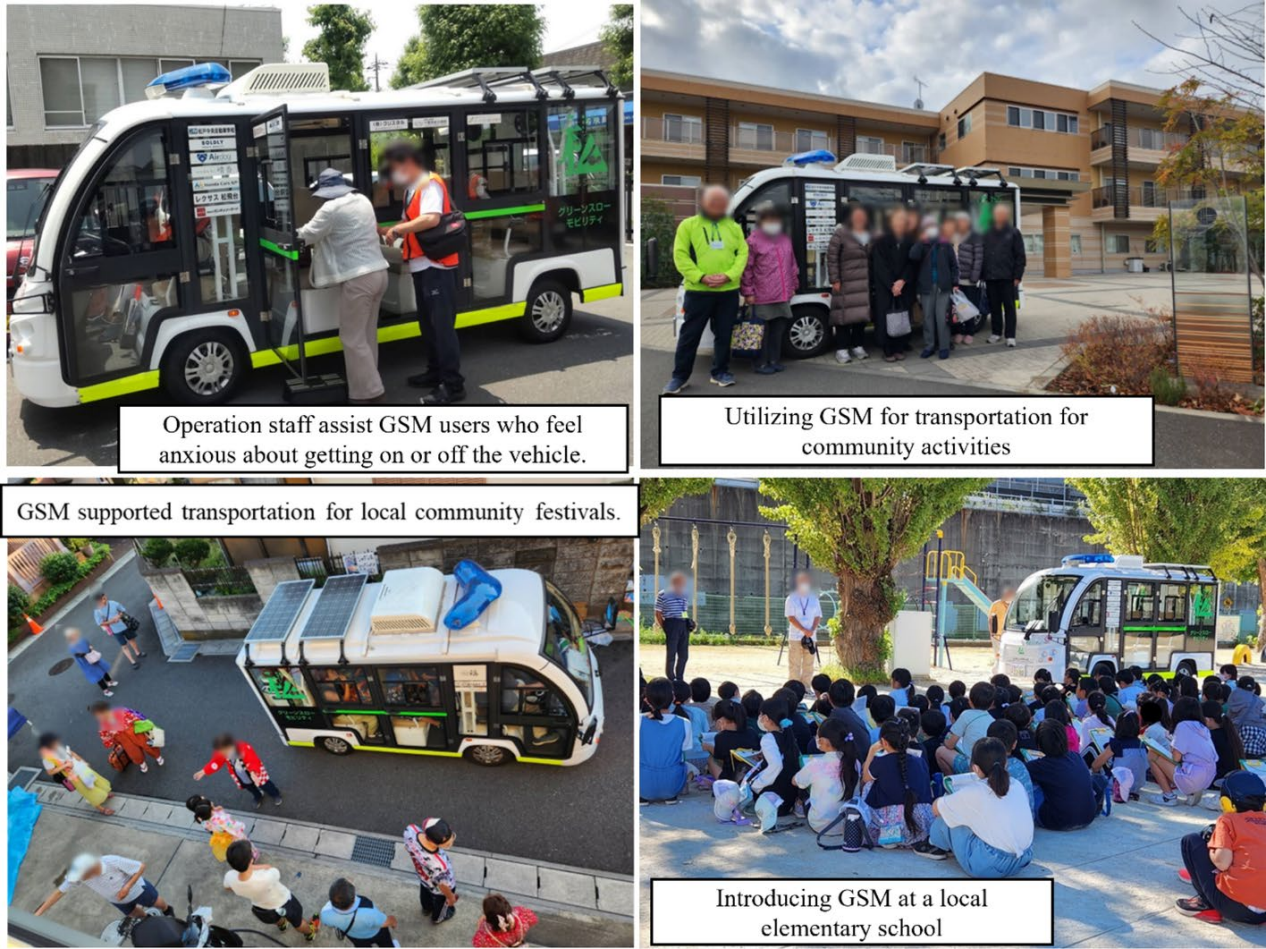
Green slow mobility operating in Matsudo city

Matsudo City, located in the Tokyo metropolitan area, covers 61 km^2^ and had a population of 497,993 in 2023, with 25.9% aged 65 years or older [32]. Figure 1 shows scenes from GSM operating in Matsudo City.During our study period, GSM operated in two residential districts in Matsudo City: Kawarazuka and Koganehara. In both areas, local residents managed the operations of GSM, and passengers used the service free of charge. Matsudo City conducted GSM trial operations for approximately four weeks in 2019 and approximately eight weeks in 2021. Kawarazuka cooperated in both trials, while Koganehara cooperated in the 2021 trial. Following these trial operations, Matsudo City fully introduced GSM in two areas starting in 2022. Kawarazuka belongs to Toubu District (population: 49,415; aging rate: 21.1%). Kawarazuka is a hilly residential area with many narrow streets that do not allow access to conventional route buses. In Kawarazuka, GSM provided two round trips per day on weekdays (one in the morning and one in the afternoon) and one trip on Saturday mornings. The route connected to a community center, a supermarket, and a local park. Each GSM round trip in Kawarazuka was 3 km and took about 30 min. The GSM operation team in Kawarazuka consisted of 59 members, including 36 drivers and 23 driver assistants. The total number of GSM users at Kawarazuka in fiscal 2023 was 2,635. Koganehara has 27,395 residents and an aging rate of 32.1%, the highest in Matsudo City. Koganehara is a flat area but functions as a transit desert due to limited access to public bus services. In this district, GSM provided six round trips per day on weekdays, with three trips in the morning and three in the afternoon. The route connected to a municipal branch office, a supermarket, a nearby train station, and a community center. On Wednesday afternoons, the operators provided the service by reservation. Each GSM round trip in Kawarazuka was 4 km and took about 40 min. The GSM operation team in Koganehara consisted of 96 members, including 87 drivers and 9 driver assistants. The total number of GSM users at Koganehara in fiscal 2023 was 3,545.

### Data collection and participants

**Fig. 2:**
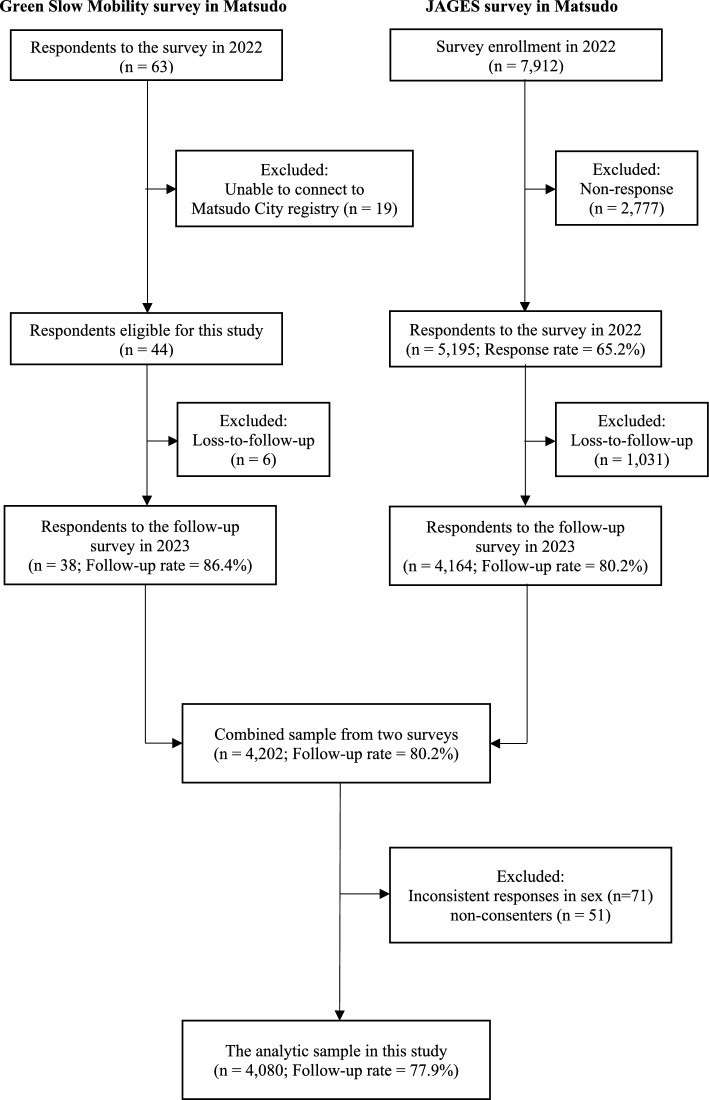
Participant flow for analytic sample

Figure 2 shows a detailed flowchart of the participant selection. In this study, we included participants from two sources: the GSM Survey and the JAGES survey. For the GSM survey in 2022, officials from Matsudo City asked GSM users in two areas where GSM was in operation to respond to a questionnaire. Participants placed complete questionnaires in collection boxes located in each area. We received responses from 63 individuals and successfully linked 44 of them to the Matsudo City registry. In 2023, we conducted a follow-up postal survey with these respondents and obtained 38 responses. For the JAGES Survey in 2022, we mailed questionnaires in 2022 to a random sample of 7,912 older residents across Matsudo City and received responses from 5,195 individuals. In 2023, we conducted a follow-up survey and obtained responses from 4,164 participants. We merged respondents who completed both the baseline and follow-up surveys from both sources. We excluded individuals who provided inconsistent answers regarding gender and those who did not consent to participate. The final analytic sample included 4,080 participants (Fig.2).

The Ethics Committee of the Japan Agency for Gerontological Evaluation Study approved this study (2023-03). We informed participants that by checking the consent box and returning the questionnaire, they agreed to participate in the study.

### Outcome variables

We hypothesized that GSM use is associated with higher frequency of outings and greater social support, as well as fewer depressive symptoms [28, 29]. Based on this hypothesis, we set depression as the primary outcome and frequency of outings and the number of social supports as secondary outcomes.

We assessed depressive symptoms using the 15-item Geriatric Depression Scale (GDS-15) [33], a validated tool for screening depression among older adults [34]. We treated the GDS-15 score as a continuous variable ranging from 0 to 15. Higher GDS-15 scores indicate more severe depressive symptoms [34].

We assessed frequency of outings by asking participants, “How often do you go out? (This includes activities such as going to the fields, visiting neighbors, shopping, or attending medical appointments).” Response options included: “5 or more times per week,” “4 times per week,” “2–3 times per week,” “once a week,” “1–3 times per month,” “a few times per year,” and “never.” We coded responses on a 0–6 scale (0 = never, 6 = 5 or more times per week), and treated the variable as continuous in our analyses [35]. This means that higher values indicate more frequent outings [35].

We assessed the number of social supports using emotional and instrumental support, both received and provided. To measure emotional support, we asked participants whether they had someone who listened to their concerns or complaints (received support), and whether they themselves listened to someone else's concerns or complaints (provided support). For instrumental support, we asked whether participants had someone who could care for them if they were ill and bedridden for several days (received support), and whether they themselves provided such care to others under similar conditions (provided support). We calculated a total score ranging from 0 to 4 by summing the number of affirmative responses across the four items [36].

### Exposure variable

We assessed our exposure, GSM use, during the follow-up wave conducted in 2023. We assessed GSM use by asking participants, “Since January 2023, how often have you used Green Slow Mobility (GSM)?” Response options included: “4 or more times per week,” “2–3 times per week,” “once per week,” “1–3 times per month,” “a few times per year,” and “never.” We defined GSM users as those who reported using GSM at least once per month, and treated this variable as binary in our analysis, with non-users serving as the reference group.

### Covariates

Based on previous studies examining the associations between public transportation, GSM, and health [14, 25, 26], we selected 13 covariates from the 2022 baseline survey as potential confounders of the association between GSM use and health-related outcomes. These covariates including gender (men or women), age (continuous), educational attainment (≥ 10 years or ≤ 9 years), household equivalized income (continuous), employment (≥ 2–3 days per week or ≤ 1 day per week), activities of daily living (independent or dependent), living arrangement (living alone or living with others), marital status (married or not married), self-reported medical illness (presence or absence), monthly participation in sports or volunteer groups (yes or no), depressive symptoms (GDS-15, continuous), frequency of outings (continuous), and the number of social supports (continuous).

### Statistical analysis

We presented continuous variables as means and standard deviations (SDs), and categorical variables as numbers and percentages in the descriptive analysis.

In this study, we used the augmented inverse probability weighting (AIPW) method [37, 38] to estimate the average treatment effect (ATE) of GSM use. AIPW is a doubly robust approach that combines features of both regression-based and inverse probability weighting estimators [37, 38]. This method requires correct specification of either the propensity score model or the outcome model, but not both [37]. We compared absolute standardized mean differences (ASMDs) before and after adjustment to assess covariate balance between GSM users and non-users. We considered an ASMD less than 0.25 to indicate an acceptable balance [39].

We estimated propensity scores using a logistic regression model that included 13 baseline covariates, and we applied the same covariates in the outcome regression model. To estimate the ATE, we used a weighted linear regression model to compare expected outcomes between the GSM group and the non-GSM group. We reported the ATE along with its 95% confidence interval (CI) and p-value. We defined outcomes using the 2023 follow-up survey and adjusted all analyses for baseline characteristics, including baseline values of the outcome variables measured in 2022.

To account for multiple testing and reduce the risk of Type I errors associated with evaluating three outcomes simultaneously, we applied the Bonferroni correction [40]. We divided the nominal significance level (α = 0.05) by the number of outcomes (n = 3), yielding a more stringent threshold for statistical significance (*p* < 0.0167).

The self-administered questionnaires contained missing data. We imputed missing values using random forest imputation, an iterative method based on random forests that performs multiple imputation by averaging results from many unpruned classification or regression trees [41]. Supplementary Table 1 details the number and proportion of missing values.

We conducted three additional analyses. First, we ran conventional linear regression models adjusted for the same covariates to assess the robustness of the AIPW estimates. Second, to explore potential pathways linking GSM use and social support, we conducted additional analyses that decomposed social support into two conceptually distinct subscales: support received and support provided. We performed these analyses using both AIPW and conventional linear regression models. Third, to evaluate the robustness of the estimated associations against unmeasured confounding, we calculated E-values for each exposure–outcome association (VanderWeele and Ding 2017). E-values represent the minimum strength of association that an unmeasured confounder would need to have on the risk ratio scale with both the exposure and the outcome, conditional on the measured covariates, to fully explain the observed association. We calculated E-values based on the point estimates of the average treatment effect (ATE) derived from the augmented inverse probability weighting models. In addition, we calculated E-values for the lower bounds of the 95% confidence intervals to provide a more conservative assessment of robustness.

We performed missing data imputation using R, version 4.2.1. We conducted all other analyses using Stata 17/IC (StataCorp, College Station, TX, USA).

## Results

Table 1 shows the baseline characteristics taken from the 2022 survey (n = 4,080). Compared to non-users (n = 4,052), GSM users (n = 28) were more likely to be older, female, have higher educational attainment and household equivalized income, be unemployed, live alone, and participate in sports and volunteer groups at least once a month. After applying PSW, all ASMD were below 0.25 (Supplementary Table 2). This result indicates that we achieved a good covariate balance between the two groups [39].

**Table 1:** Baseline characteristics from 2022 survey (n = 4,080)

Baseline characteristics	Green slow mobility	
	Non-user	User
	n = 4,052	n = 28
Age (years), mean (SD)	75.7 (6.0)	78.7 (5.1)
Gender (Women), n (%)	2,054 (51.1)	16 (57.1)
Education (≤ 9 years), n (%)	555 (13.7)	3 (10.7)
Household equivalized income (million yen), mean (SD)	2.570 (1.618)	2.968 (1.682)
Employment (≥ 2–3 days per week), n (%)	1,009 (24.9)	3 (10.7)
Activities of daily living (independent), n (%)	3,930 (96.9)	27 (96.4)
Living alone, n (%)	723 (17.8)	6 (21.4)
Marital status (Married), n (%)	2,896 (71.4)	20 (71.4)
Self-reported disease, n (%)	3,202 (79.0)	22 (78.5)
Monthly participation in sports groups, n (%)	1,260 (31.1)	13 (46.4)
Monthly participation in volunteer groups, n (%)	490 (12.0)	11 (39.2)
Depressive symptoms, mean (SD)	2.8 (2.8)	2.6 (2.1)
Frequency of outings, mean (SD)	5.2 (1.0)	5.2 (0.8)
Number of social supports, mean (SD)	3.6 (0.7)	3.7 (0.5)

SD, standard deviation

Table 2 presents the ATEs for health-related outcomes. We estimated ATEs using AIPW as the main analytical method and included results from conventional linear regression models as an additional analysis. We found that GSM use was associated with lower depressive symptoms (ATE = − 1.39; 95% CI: − 1.94 to − 0.83; *p* < 0.001), higher frequency of outings (ATE = 0.60; 95% CI: 0.43 to 0.77; *p* < 0.001), and greater the number of social supports (ATE = 0.25; 95% CI: 0.12 to 0.38; *p* < 0.001). We also estimated the average potential outcomes among GSM users, which were 3.01 for depressive symptoms, 5.14 for outing frequency, and 3.60 for the number of social supports. The results of conventional linear regression analysis in additional analysis also supported the main analysis.

**Table 2:** Associations between Green slow mobility use and health-related outcomes

Outcomes in 2023	Augmented inverse probability weighting				Linear regression			
	ATE^a^	95%CI		*p*	B	95%CI		*p*
Depressive symptoms	− 1.39	− 1.98	− 0.83	< 0.001 **	− 0.73	− 1.20	− 0.26	0.005 **
Frequency of outing	0.60	0.43	0.77	< 0.001 **	0.45	0.05	0.85	0.028 **
Number of social supports	0.25	0.12	0.38	< 0.001 **	0.22	0.16	0.28	< 0.001 **

ATE, average treatment effect; *B*, unstandardized coefficients; CI, confidence interval

^a^ATE represents the difference between the average outcome for Green Slow Mobility use and the average outcome for the same group if they had not used Green Slow Mobility

^*^p < .05 before Bonferroni correction; **p < .05 after Bonferroni correction (the *p*-value cutoff for Bonferroni correction is *p* = .05/3 outcomes = *p* < .0167)

Supplementary Table 3 presents additional analyses decomposing social support into received and provided. In conventional linear regression analyses, GSM users reported a greater number of both received and provided social supports than non-users. In the AIPW models, GSM use was associated with greater number of both received and provided social support; however, after Bonferroni correction, the association with provided social support did not meet the adjusted significance threshold.

Supplementary Table 4 presents the calculated E-values, which suggest that the observed associations between GSM use and health-related outcomes were relatively robust to potential unmeasured confounding. For example, to fully account for the association between GSM use and depressive symptoms, an unmeasured confounder would need to be associated with both the exposure and the outcome by a risk ratio of at least 6.55 each, conditional on the measured covariates. To shift the lower bound of the confidence interval to include the null, the confounder would need to have a minimum risk ratio association of 3.68 with both the exposure and the outcome.

## Discussion

Our study provided empirical evidence on the associations between GSM use and health-related outcomes among older adults in Matsudo City, an urban area in Japan. Compared to non-users, GSM users showed fewer depressive symptoms, the primary outcome. In addition, secondary outcomes, including frequency of outing and the number of social supports, were higher among GSM users than non-users. Our results are consistent with the hypothesis that GSM use is associated with higher frequency of outings and greater social support, as well as fewer depressive symptoms.

Our findings align with previous studies demonstrating the positive impact of transportation on health [14, 15] and with earlier evaluations of GSM implementation [25, 26]. These evaluations indicated that GSM users experienced subjective improvements in communication and mutual support [26]. Community-led GSM operations may foster a sense of familiarity and openness, facilitating communication between users and staff. GSM is primarily used for shopping for daily necessities [25]. In Matsudo City, the GSM route provided regular access to several supermarkets. Incorporating GSM into daily routines may enhance not only mobility but also opportunities for social interaction through shopping.

Because the GSM operation in Matsudo City was community-based, potential health benefits may extend beyond users to include operational staff. GSM users showed high volunteer participation and low employment at baseline, indicating community engagement. The observation that GSM users reported higher levels of both received and provided social support supports the possibility that participation in GSM involves reciprocal social engagement. Previous studies have demonstrated that active engagement in social roles protects against depression [42] and dementia [43]. Operational staff may serve as ongoing sources of support through their daily interactions with users. Our findings may reflect not only the effects of GSM as a transportation service but also the influence of volunteer activities related to GSM operations. Due to the limited sample size, our study could not distinguish between users and staff in the analysis. Future studies should explicitly differentiate passengers from those involved in GSM operations to clarify the distinct potential health effects of transportation use and volunteer participation.

Policymakers continue to grapple with the sustainability of public transportation, with financial constraints representing the primary challenge [19, 20]. Introducing community-based GSM may offer a viable solution. Although initial investments are required for vehicle procurement and maintenance, GSM has the potential to deliver benefits that reach beyond mobility, including improvements in the health of older adults [25]. These broader effects may justify the cost over the long term. Sustaining community-based GSM operations depends heavily on the existing level of social capital within the community. Matsudo City had already promoted social capital through its urban healthy aging strategy [31] before GSM was introduced. Policymakers should focus on strengthening community networks and social infrastructure to support the successful implementation and sustainability of GSM.

Our study has six limitations. First, we included only a small number of GSM users. This limited our ability to perform additional subgroup analyses and assess the robustness of the findings in greater detail. We addressed this limitation by applying both conventional linear regression and augmented inverse probability weighting models. Second, we could not analyze associations by usage frequency or distinguish between users and operational staff. Third, although we adjusted for a wide range of baseline covariates, we could not eliminate the possibility of unmeasured confounding. To address this concern, we calculated E-values to assess the potential impact of such confounding. Fourth, we analyzed only participants who completed both baseline and follow-up surveys. This may have introduced selection bias. Among older adults, those who dropped out of study tended to have poorer health status and behaviors [44]. Therefore, the final sample may have been relatively healthier, which could lead to an underestimation of the associations. Fifth, although our study used two-wave data, GSM use and health outcomes were assessed concurrently at follow-up. Therefore, temporal precedence cannot be established, and reverse causality remains possible. Further longitudinal data preparation and analysis will be necessary in the future. Finally, we conducted the study in two local communities within a single Japanese municipality. This limits the generalizability of our findings. In addition, Japan has relatively high public safety and strong community infrastructure. These contextual factors may differ from those in other countries and should be considered when applying the findings to other settings.

## Conclusion

We demonstrated that GSM use and volunteer activities related to GSM operation were associated with fewer depressive symptoms, the primary outcome, and with higher outing frequency and social support, the secondary outcomes, among older adults in Matsudo City. Our findings suggest that integrating community-based transport models, such as GSM, into daily life can support not only physical mobility but also psychosocial health. We recommend that policymakers consider GSM as a viable strategy to promote healthy aging, particularly in urban areas with aging populations.

## Supplementary Information

Below is the link to the electronic supplementary material.

Supplementary file1 (DOCX 36 kb)

## Data Availability

Data are available upon reasonable request and approval from the Japan Agency for Gerontological Evaluation Study and Matsudo City. Researchers must also register with the Matsudo City researcher registry.
