# Supplementary material for: The association between community-based transportation use and depressive symptoms among older adults in Japan
Source: Discov Ment Health. 2026 Feb 23;6(1):50. doi: 10.1007/s44192-026-00395-7 (PMC13031565; doi:10.1007/s44192-026-00395-7)
Supplement: Supplementary file 1 — Supplementary file1 (DOCX 36 kb) [file 44192_2026_395_MOESM1_ESM.docx]

Supplementary Table 1. Number (Percentage) of missing values

|  | Variables | N (%) |
| --- | --- | --- |
|  | Baseline Survey in 2022 |  |
|  | Age | 0 (0) |
|  | Gender | 0 (0) |
|  | Education | 106 (2.6) |
|  | Household equivalized income | 387 (9,4) |
|  | Employment | 281 (6,8) |
|  | Activities of daily living | 49 (1.2) |
|  | Living alone | 111 (2.7) |
|  | Marital status | 29 (0.7) |
|  | Self-reported disease | 129 (3.1) |
|  | Monthly participation in sports groups | 125 (3.0) |
|  | Monthly participation in volunteer group | 149 (3.6) |
|  | Depressive symptoms | 498 (12.2) |
|  | Frequency of outing | 91 (2.2) |
|  | Number of social supports | 17 (0.4) |
|  | Follow up Survey in 2023 |  |
|  | Depressive symptoms | 480 (11.7) |
|  | Frequency of outing | 96 (2.3) |
|  | Number of social supports | 33 (0.8) |
|  | Green Slow Mobility Use | 743 (18.2) |

Supplementary Table 2. Absolute standardized mean differences before and after weighting for Green Slow Mobility user and non-use

|  |  | Absolute standardized mean differences | |
| --- | --- | --- | --- |
|  |  | Before weighting | After weighting |
|  | Age | 0.51 | 0.08 |
|  | Gender | 0.11 | -0.02 |
|  | Education | -0.09 | 0.20 |
|  | Household equivalized income | 0.24 | 0.15 |
|  | Employment | -0.37 | 0.17 |
|  | Activities of daily living | 0.03 | 0.05 |
|  | Living alone | 0.08 | -0.08 |
|  | Marital status | 0.00 | -0.23 |
|  | Self-reported disease | 0.01 | 0.09 |
|  | Monthly participation in sports groups | 0.31 | 0.18 |
|  | Monthly participation in volunteer group | 0.64 | 0.23 |
|  | Depressive symptoms | -0.10 | -0.23 |
|  | Frequency of outing | -0.10 | -0.13 |
|  | Number of social supports | 0.14 | 0.01 |

Supplementary Table 3 Associations between Green Slow Mobility use and Number of social supports

| Outcomes in 2023 | Augmented inverse probability weighting | | | | Linear regression | | | |
| --- | --- | --- | --- | --- | --- | --- | --- | --- |
|  | ATE^a^ | 95%CI | | p | B | 95%CI | | p |
| Number of received social supports | 0.10 | 0.09 | 0.12 | <0.001 ** | 0.08 | 0.04 | 0.12 | 0.001 ** |
| Number of provided social supports | 0.14 | 0.01 | 0.27 | 0.029 * | 0.14 | 0.07 | 0.21 | 0.001 ** |

Abbreviations: ATE, average treatment effect; *B*, unstandardized coefficients; CI, confidence interval.

^a^ ATE represents the difference between the average outcome for Green Slow Mobility use and the average outcome for the same group if they had not used Green Slow Mobility.

* p < .05 before Bonferroni correction; ** p < .05 after Bonferroni correction (the p-value cutoff for Bonferroni correction is p = .05/2 outcomes = p < .025).

Supplementary Table 4 Robustness to unmeasured confounding (E-values) of associations between Green Slow Mobility use and health-related outcomes.

| Outcomes in 2023 | | E-values | |
| --- | --- | --- | --- |
|  |  | Point Estimate | CI limit |
|  | Depressive symptoms | 6.55 | 3.68 |
|  | Frequency of outing | 2.85 | 2.32 |
|  | Number of social supports | 1.84 | 1.48 |

Abbreviations: CI, confidence interval.
